# MEASURING GOALS, PRIORITIES, AND PREFERENCES: REFORM EFFORTS FOR TRANSPARENCY AND EMPOWERMENT

**DOI:** 10.1093/geroni/igad104.0961

**Published:** 2023-12-21

**Authors:** Tara McMullen, Anna Fisher

**Affiliations:** U.S. Department of Veterans Affairs , Sykesville, Maryland, United States; Hillcrest Health Services, Bellevue, Nebraska, United States

## Abstract

Longstanding evidence suggests that the goals, preferences, and priorities (GPP) of individual nursing facility residents may not be adequately addressed during episodes of care. Aligning care with residents’ GPP represents an opportunity to improve outcomes and reduce disparities associated with race, ethnicity, gender orientation, and other characteristics of the resident population. The 2022 National Academies of Sciences, Engineering, and Medicine (NASEM) report recommended improvement in quality measures under the domain of resident and family experience, along with reporting of these measures on Care Compare to improve shared decision-making and transparency. The subcommittee on quality improvement and measurement is focused primarily on measures of GPP as a useful addition to CMS’ plans to expand the Quality Reporting Program (QRP) measure set, which was signaled in the recently issued Skilled Nursing Facility Prospective Payment System (SNF PPS) rule. In this presentation, committee members will discuss the identification and testing plan for one or more resident experience of care measures that assess the experience of care specific to processes of care and the resident’s GPP. This presentation will also discuss the development of a care concordant measure, assessing the relationship between the care provided to the resident and the resident GPPs.

